# Reply to “Urine-based detection of HPV for cervical cancer screening: towards clinical implementation”

**DOI:** 10.1128/jcm.00162-26

**Published:** 2026-03-16

**Authors:** Sharmila Manjeshwar, Noah Kojima, Eric Tsang, David Pereira, Emmanuel Silva, Ha Duong, Felix Chao, Daniel R. Marshak, Ricky Y. T. Chiu, Jeffrey D. Klausner

**Affiliations:** 1 Phase Scientific Americas, Garden Grove, California, USA; 2 Department of Medicine, University of California 8783, Los Angeles, California, USA; 3 Phase Scientific International Ltd, Hong Kong, China; 4 Keck School of Medicine, University of Southern California 5116, Los Angeles, California, USA; Mayo Clinic Minnesota, Rochester, Minnesota, USA

## REPLY

We thank the authors for their interest in our report describing a novel, urine-based test for the detection of cervical human papillomavirus infection (HPV) (1). It is well understood that early detection of high-risk HPV infection is important to prevent cervical cancer, and we believe that urine-based HPV testing is a promising non-invasive alternative to traditional cervical cancer screening approaches.

We agree with the authors’ comments regarding the potential impact of urine collection volume on analytical sensitivity, and that larger first-void volumes may dilute HPV DNA. We want to highlight that our proprietary PHASiFY technology-based extraction method is uniquely designed to concentrate DNA from large sample input volumes, thereby counteracting the dilution effects that are seen when traditional DNA extraction methods are used for large urine volumes (i.e., over 30 mL). As a result, variations in collection volume do not impact the ability of this technology to extract nucleic acids as long as the concentrations are at or above the lower limit of detection (15–30 copies/mL) as determined in our analytical studies.

We would like to clarify that the Phase HPV Urine Test is standardized for 40 mL urine sample collection for routine patient testing in the Clinical Laboratory Improvement Amendments (CLIA) laboratory. This standardized volume is supported by product development and verification studies to be the optimal volume of sample that retains the analytical sensitivity/low limit of detection and high overall percent agreement with the reference method. However, in the validation study described in this report, the rationale for collecting up to 100 mL was to have sufficient volume for two separate DNA extractions (40 mL urine each) at two different time points from the same patient sample. The results from the stability study indicate that in this “worst-case scenario,” the samples of up to 100 mL still demonstrated high concordance with the reference method; and HPV detection between the two time points was robust with a non-significant difference on Ct values from the one sample collection (1).

We appreciate the authors’ comments regarding platform compatibility, affordability, and standardized detection systems in clinical laboratories. Indeed, the core elements of the workflow for the Phase HPV Urine Test—from urine stabilization, nucleic acid extraction and concentration, and target amplification—are standard technologies that can be implemented in any clinical laboratory. Specifically, for HPV detection, we developed and employed a standard fluorescent probe-based PCR test that is performed on a commercially available real-time PCR platform (Thermo Fisher QuantStudio). The next-generation sequencing (NGS) test performed in our study was used only as an independent test to verify and adjudicate the results of the PCR test to demonstrate assay performance and is not part of the routine workflow of the Phase HPV Urine Test.

We also appreciate the authors’ comments on other diagnostic approaches, such as isothermal amplification methods for HPV detection, that might provide simpler and more affordable alternatives in low- or middle-income countries. We acknowledge the applicability of such technologies, and we are investigating this aspect as part of future product development activities.

Finally, we share the authors’ view that broader technical considerations, regulatory validation, standardized protocols for urine sample collection and testing, as well as patient-centered implementation strategies are critical for successful translation and implementation of urine-based HPV testing into routine clinical practice. The analytical validation study presented in this report (1) addresses some of these aspects and demonstrates that the Phase HPV Urine Test is a robust, reliable, and standardized workflow that can be implemented in the clinical laboratory. In addition, we have several ongoing clinical studies that are focused on multi-site clinical validation with histology-confirmed endpoints, refinement of clinical cycle threshold cutoffs as well as usability assessments for self-collection and laboratory workflows. An initial study in an enriched population has been completed and published (2), whereas a second, larger study in a screening population with 17,000 patients is still ongoing.

In conclusion, we agree that robust guidelines and clear communication of benefits and limitations are central to building confidence among clinicians, laboratorians, and patients for urine-based HPV tests. Our studies are intended to generate data aligned with regulatory expectations for *in vitro* diagnostic devices and evidence-based guidelines for cervical cancer screening.

We believe that non-invasive, scalable sensitive screening tools, such as urine, have the potential to expand access to cervical cancer prevention, particularly among underserved and under-screened populations, and it is necessary that the field moves towards practical solutions for cervical cancer screening parity.
